# Demographic factors associated with myopia knowledge, attitude and preventive practices among adults in Ghana: a population-based cross-sectional survey

**DOI:** 10.1186/s12889-023-16587-7

**Published:** 2023-09-04

**Authors:** Uchechukwu L. Osuagwu, Stephen Ocansey, Antor O. Ndep, Sylvester Kyeremeh, Godwin Ovenseri-Ogbomo, Bernadine N. Ekpenyong, Kingsley E. Agho, Edgar Ekure, Khathutshelo Percy Mashige, Kelechi C. Ogbuehi, Tuwani Rasengane, Nana Darkoah Nkansah, Kovin Shunmugan Naidoo, Centre for Eyecare & Public Health Intervention Initiative (CEPHII)

**Affiliations:** 1 Bathurst Rural Clinical School (BRCS), School of Medicine, Western Sydney University, PO Box 9008, Bathurst, NSW 2795 Australia; 2 African Vision Research Institute, Discipline of Optometry, University of KwaZulu-Natal, Westville Campus, Durban, 3629 South Africa; 3 Department of Optometry and Vision Science, School of Allied Health Sciences, College of Health and Allied Sciences, University of Cape Coast, Cape Coast, Ghana; 4 Health Education & Health Promotion Unit, Department of Public Health, Faculty of Allied Medical Sciences, College of Medical Sciences, University of Calabar, Calabar, Cross River State Nigeria; 5 Department of Optometry and Visual Science, College of Science, Kwame Nkrumah University of Science & Technology (KNUST), Kumasi, Ghana; 6 Department of Optometry, Centre for Health Sciences, University of the Highlands and Islands, Inverness, IV2 3JH UK; 7 Epidemiology and Biostatistics Unit, Department of Public Health, University of Calabar, Calabar, Nigeria; 8 School of Health Sciences, Western Sydney University, Campbelltown, NSW 2560 Australia; 9 Roberts Eyecare Associates, Vestal, NY USA; 10 Department of Medicine, Dunedin School of Medicine, University of Otago, Dunedin, New Zealand; 11 Department of Optometry, University of the Free State and Universitas Hospital, Bloemfontein, South Africa; 12 Koforidua Regional Hospital, Koforidua, Eastern Region Ghana; 13 School of Optometry and Vision Science, University of New South Wales, Sydney, NSW Australia

**Keywords:** Myopia, Knowledge, Attitude, Preventive practices, Sub-Saharan Africa, Ghana

## Abstract

**Purpose:**

Knowledge, positive attitude and good preventive practices are keys to successful myopia control, but information on these is lacking in Africa. This study determined the KAP on myopia in Ghana.

**Methods:**

This was a population-based cross-sectional survey conducted among adults (aged 18 years and older) living across 16 regions of Ghana between May and October 2021. Data on socio-demographic factors (sex, age, gender, level of education, working status, type of employment, monthly income, and region of residence), respondents’ awareness, and knowledge, attitude and preventive practices (KAP) about myopia were collected. Composite and mean scores were calculated from eleven knowledge (total score = 61), eight attitude (48), and nine preventive practice items (33). Differences in mean scores were assessed using one-way analysis of variance (ANOVA) and standardized coefficients (β) with 95% confidence intervals (CI), using multiple linear regression to determine the associations between the dependent (KAP) and demographic variables.

**Results:**

Of the 1,919 participants, mean age was 37.4 ± 13.4 years, 42.3% were aged 18–30 years, 52.6% were men, 55.8% had completed tertiary education, and 49.2% had either heard about myopia, or accurately defined myopia as short sightedness. The mean KAP scores were 22.9 ± 23.7, 33.9 ± 5.4, and 22.3 ± 2.8, respectively and varied significantly with many of the demographic variables particularly with age group, region, marital status, and type of employment. Multiple linear regression analyses revealed significant associations between region of residence and knowledge (β =—0.54, 95%CI:-0.87, -0.23, *p* < 0.001), attitude (β =—0.24, 95%CI:-0.35,-0.14, *p* < 0.001) and preventive practices (β = 0.07, 95%CI: 0.01, 0.12, *p* = 0.015). Preventive practices were also associated with type of employment (self-employed vs employee: β = 0.25, 95%CI: 0.15, 4.91, *p* < 0.05). Knowledge scores were significantly higher in those who lived in the Greater Accra (39.5 ± 18.5) and Eastern regions (39.1 ± 17.5) and lower among those who lived in the Upper West region (6.4 ± 15.6). Government employees and those with tertiary education had significantly higher mean knowledge scores compared with non-government employees (β = 4.56, 95%CI 1.22, 7.89, *p* = 0.007), and those with primary/no education (β = 18.35, 95%CI: 14.42, 22.27, *p* < 0.001).

**Conclusion:**

Ghanaian participants had adequate knowledge of myopia but showed poor attitude and low preventive practices, which varied significantly between regions and were modified by socio-demographic factors. Further research into how education can be used to stimulate Ghanaians’ engagement in preventive practices is needed.

**Supplementary Information:**

The online version contains supplementary material available at 10.1186/s12889-023-16587-7.

## Introduction

Globally, the prevalence of myopia remains progressively high [1] and is projected to affect 50% of the world’s population by 2050, with about 10% expected to have high myopia (≥ 5.00D) [1]. Myopia prevalence varies depending on race and geographical region [2] with a reported prevalence of 80–90% among young adults in East Asia and 10–20% in Southeast Asia [3]. In Africa, the crude prevalence of myopia was estimated at 4.7% (95% CI, 3.9–5.7) [4], and up to 33.5% in adults [5]. In Ghana, the prevalence of myopia has been reported to be about 2% among school children [4], 25.1% in teenage secondary school students [6] and between 29.2%—54.1% in clinic-based studies [7, 8]. Judging by the trend in global epidemiology of myopia [9], the myopia situation in Africa is expected to increase due to inadequate access to eye and vision care services.

Myopia is known to have health, social and economic implications [6–8]. In 2015, the global loss of productivity from visual impairment due to uncorrected myopia was estimated at $244 billion [6]. Among older Chinese adults, those with myopia were about 1.4 times more likely to have depressive symptoms than non-myopes [8]. Despite these, in many developing countries, myopia remain undiagnosed. If left untreated, high myopia can significantly increase the risk of several serious eye conditions and complications that can lead to blindness, so uptake of refractive services are crucial.

There is strong evidence that simple environmental and clinical factors such as increased time outdoors [9], low dose atropine eye drops [10], and optical interventions such as orthokeratology can help delay the onset and/or slow the progression of myopia [9]. The effectiveness of these clinically proven myopia interventions largely depend on compliance with management protocols and the understanding of the affected people or their guardians [11]. Notwithstanding, studies indicate that there is a lack of information about myopia, its prevention and treatment among parents/guardians of children with myopia in various places, which may impact the uptake of myopia control services. A study conducted in Ireland reported that only 46% of parents considered myopia a health risk [12] while 80% of parents in Shanghai thought myopia could be cured [13]. Among mothers in Hong Kong, Cheung et al. [14] found that more than half believed that corrective contact lenses were only meant for older children (14 years and above) [14] and not for younger ones. These results confirm the lack of knowledge about myopia among the public, necessitating the need for further research in the areas, especially in low resourced environments.

Hence, the purpose of this study was to determine the knowledge, attitude, and preventive practices of adults toward myopia in Ghana. This study will provide evidence on the level of knowledge about myopia, the attitude, and practices in relation to myopia among an African population, which can be used to provide a framework for further research in myopia control. Also, it will help design myopia educational resources for the public and assist in developing policies and planning myopia control programs in similar environments.

## Methods

### Study design

This was a population-based online cross-sectional survey conducted in Ghana, between May and October 2021 among eligible adults aged 18 years and above who provided consent to participate in the study.

### Study setting and population

Ghana has a youthful population with an estimated national literacy rate of 69.8%. About 22% of the total population of nearly 31 million [50.7% women] are adolescents (16), the age during which myopia becomes progressively worse, before stabilising in early adulthood.

The Government lunched the National Eye Health Programme (NEHP) in 2000 for the elimination of avoidable blindness by the year 2020, in response to the global initiative VISION 2020-The Right to Sight. Prior to the launch, eye care provision was largely limited to Accra and other urban areas, leaving majority of Ghanaians to either self-medicate or seek help from traditional healers. Eye care services in Ghana have seen a lot of progress through collaboration and partnership with the World Health Organization and various non-governmental organizations (NGOs). Despite the progress in eye care, the prevalence of myopia is increasing with about 2% of Ghanaians reported to be either blind or have a severe visual impairment [15]. Data available also indicate that 67.74% of persons who were blind dwell in areas where there are no programs of blindness prevention or treatment intervention going on, while 32.26% live in areas where some programs of intervention are being implemented. Most vision-related research [4, 16–18] in Ghana are focused on the prevalence and risk factors of refractive errors, visual impairment, other ocular morbidities and their related socio-economic issues.

### Sample size

The minimum sample size was determined using published tables and calculated based on the estimated prevalence of 50% of myopia knowledge among the population, at a precision level of 5% and a 95% confidence interval. For a population above 100,000, it was determined that a minimum sample size of 400 was required to achieve 80% power to detect significant differences [19]. However, a total of 1,919 adult respondents from the online surveys participated in the study.

### Questionnaire

Data was collected using a 28-item Likert scale, a self-administered questionnaire developed by members of the African Eye and Public Health Research Initiative (AEPRI) and was standardized through a pilot study in which 20 individuals aged 18 years or more were randomly selected to complete the questionnaire. The pilot was to determine how participants would interpret the questionnaire and whether there was a need for any amendments. The pilot study also checked for typographical errors, inappropriate diction, and eliminate ambiguous or misleading items. After the pilot phase, all ambiguous questions were rephrased, inappropriate diction was reworded, and all identified errors were corrected. To achieve a reliable questionnaire, this study used Cronbach’s Alpha which is the most popular model to determine the correlation among the structured items and to assess the internal consistency of the questionnaire that is made up of multiple Likert-type scale items. The overall Cronbach’s Alpha values based on the standardized items were Knowledge (0.85), Altitude (0.70), and Practices (0.76). Generally, reliability coefficients of 0.70 are considered acceptable or reliable, therefore, there is much confidence to conclude that the structured items of the instrument were sufficiently reliable to measure myopia KAP. Data from those who participated in the pilot study were not included in the final analysis.

The final questionnaire shown in Supplementary Table 1 (Table-S1) comprised five sections including the independent variables of socio-demographic factors [sex, age, gender, level of education, working status, type of employment, monthly income, region of residence], and respondents’ awareness of myopia. Knowledge about myopia (11 items), attitudes toward myopia (8 items) and the respondents’ preventive practices against myopia (9 items) were the three dependent variables. Knowledge comprised respondents’ understanding of myopia, knowledge of risks, signs and symptoms, treatment and prevention which were framed into 11-item Likert scale questions. Attitude referred to respondents’ opinions that could influence their practices towards myopia management (positive attitude would promote positive behaviour whereas negative attitude would do otherwise). Preventive practices referred to actions that could potentially result in prevention of myopia development.

### Data collection

Data collection was collected using a self-administered anonymous online form, designed in Google form format, and was distributed via e-link by the study investigators on social media platforms (including Facebook and WhatsApp) which are easily accessible in all 16 regions of the country. Respondents enrolled voluntarily by first responding to eligibility criteria coded at the beginning of the survey, so respondents who did not meet the set criteria were automatically logged out of the survey. Exclusion criteria included persons younger than 18 years, medical and optometry students or those who worked in any eye health profession who are considered to have foreknowledge of myopia. Coverage of all regions was indicated by the responses obtained from participants. Participants were advised to complete the questionnaire only once to minimize repeated responses. Multiple submissions were also controlled by using the ‘Limit to one response’ feature of Google forms and respondents had to sign in with their email addresses in order to key in responses in Google forms. A simple search on the downloaded excel spreadsheet was done to highlight multiple email addresses and researchers deleted any multiples found. At the time of data collection, the country was still under mandatory lockdown and restrictions as a response to the COVID-19 pandemic. Therefore, it was impossible to use a paper-based survey to reach those participants who did not have access to the internet or preferred hard copies, as well as those who could not read the English Language. The duration of the survey was 15 to 20 min.

### Data analysis

The Statistical Package for Social Sciences (SPSS) Statistics for Windows, version 21. 0 (SPSS Inc., Chicago, Ill., USA) was used in data analysis after entering, cleaning and coding the data in Microsoft excel. Analysis was restricted by IP addresses such that during data cleaning, duplicate records were identified and the one with the most completed items of the questionnaire was retained and the others were deleted. Descriptive statistics including means ± standard deviations, were used for continuous variables while frequencies and proportions were used for categorical variables. Data were presented using tables and cross-tabulations to emphasize relationships. Composite scores were calculated for knowledge, attitude, and preventive practice with total scores of 61, 48 and 33 respectively, for the three dependent variables. The Likert scale questions for knowledge and attitude had six grading levels: strongly agree, somewhat agree, agree, neutral, disagree, somewhat disagree, and strongly disagree which were coded as 6, 5, 4, 3, 2 and 1, respectively for positively directed questions. That for preventive practice was graded on four levels ranked as 1 for ‘never’, 2 for ‘sometimes’, 3 for ‘often’ and 4 for ‘always’. Negative questions were reverse coded using the same grading. Scores for each item were summed for every respondent to obtain their total score for knowledge. Mean scores were then determined by dividing the total score by the total number of responses in line with the independent variables. Questions with Yes, No and Not sure responses were coded as Yes = 2, Not sure = 1 and No = 0 and analyzed using frequencies and percentages. Only the responses of those who stated that they had “heard about” myopia were included in the analysis of mean scores for all the three dependent variables.

For knowledge, all those who scored below the mean were considered to have low knowledge levels regarding myopia, and those who scored above the mean were considered to have high knowledge levels. For attitude, those who scored higher than the mean were considered to have a positive attitude that would promote positive behaviour toward myopia management, while those whose scores were below the mean were considered to have a negative attitude towards myopia management. For preventive practices, those who scored below the mean were considered to have poor myopia preventive practices, while those who scored higher than the mean were considered to have good myopia preventive practices.

Differences in mean scores were assessed using one way analysis of variance (ANOVA). Bonferroni correction was applied for multiple comparisons. Simple and multiple linear regression analyses were conducted to determine the associations between the dependent variables (knowledge, attitudes, and preventive practices) and the demographic factors. In our analysis, we checked for multicollinearity using Variance inflation facto (VIF) and homogeneity of variances. The standardized coefficients (β) with their 95% confidence intervals (CI) were calculated. A two-sided *p* value of < 0.05 was considered statistically significant.

### Ethical consideration

The research adhered to the Helsinki Declaration on research involving human subjects. Ethical approval was obtained from the Institutional Review Board of the University of Cape Coast (UCCIRB/EXT/2022/43). Participant information sheets were provided to explain the details of the study to participants and written consent forms to seek their approval before responding to the questionnaire. Participants were assured of the confidentiality and anonymity of their responses.

## Results

### Socio-demographic characteristics

Table 1 shows the demographic characteristics of the 1,919 adult participants (52.4% were men [*n* = 1006]) in this study. Most of the participants (*n* = 812, 42.3%) were aged 18—30 years, 954 (49.7%) were either married or co-habiting, and more than half, each, had acquired either tertiary or graduate level education (*n* = 1071, 55.8%), were working at the time (*n* = 1006, 52.4%), or earned less than GHȻ 1000 (~ $123USD: *n* = 986, 51.4%) per month. Of those who indicated their type of service (551 participants), 62.1% (*n* = 342) were government employees. Further demographic details are shown in Table 1.

**Table 1:** Socio-demographic characteristics of participants (*n* = 1919, otherwise indicated)

Characteristics	Frequency	Percentage (%)
**Age groups (years)**		
18–30	812	42.3
31–40	376	19.6
41–50	425	22.1
51–90	306	15.9
**Sex**		
Male	1006	52.4
Female	913	47.6
**Marital Status**		
Never married	813	42.4
Previously married^a^	152	7.9
Married/living together	954	49.7
**Work Status**		
Working	1006	52.4
Not working	913	47.6
**Employment type**		
Employee	543	54.0
Self-employed	463	46.0
***Type of service***		
Government	342	62.1
Non-government	209	37.9
**Level of education**		
Primary/No formal education	311	16.2
Secondary	537	28.0
Tertiary/Graduate school	1071	55.8
**Level of monthly income**		
Less than GHȻ 1000 (< 123USD)	986	51.4
GHȻ 1000 – GHȻ 2,999 (123-371USD)	735	38.3
GHȻ 3,000 – GHȻ 5,999 (372-744USD)	134	7.0
GHȻ 6,000 or more (≥ 745USD)	64	3.3

^a^ Divorced, widowed and separated

**Fig. 1:**
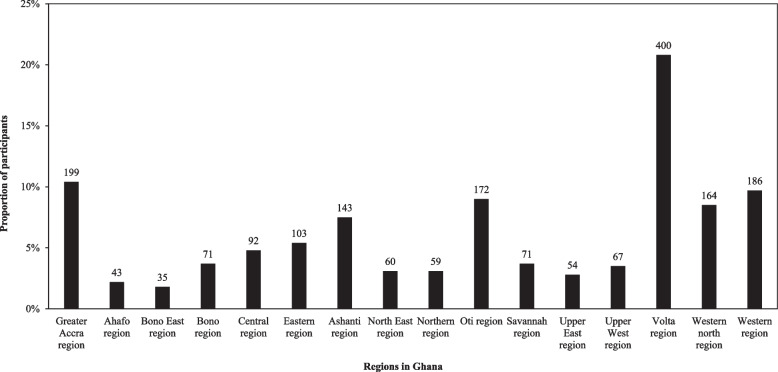
Distribution of participants by the regions in Ghana (*n* = 1,919)

Figure 1 presents the distribution of the participants by region of residence in Ghana. It shows that most participants in this study were residing in the Volta region (*n* = 400, 20.8%) followed by the region of Greater Accra (*n* = 199, 10.4%) at the time of this study.

### Awareness and knowledge of myopia

Of the 1,919 participants, 49.2% (*n* = 945) had heard about myopia and the rest (974, 50.8%) had not. Majority of those who have heard about myopia correctly defined the condition as short sightedness (*n* = 915, 96.8%) while the remaining (*n* = 30, 3.2%) defined myopia wrongly, either as longsightedness, blindness, blurry vision and others were not sure.

### Mean KAP scores by demographic variables in Ghana

Table 2 shows the mean scores for the three outcome variables in this study. The overall mean knowledge score was 45.6 ± 6.2 (range, 21 – 61). More than half the participants (*n* = 487, 51.5%) had good knowledge of myopia, with higher scores among younger adults (18—30 years old: mean score, 46.3 ± 5.9), women (46.0 ± 6.0) and those who lived in the Northern (48.0 ± 6.2), Northeast (46.8 ± 4.2), Greater Accra (46.5 ± 6.0) and Ashanti (46.3 ± 5.9) regions. Other details of demographic variations in mean knowledge scores are displayed in Table 2.

**Table 2:** Mean scores (± SD) for myopia knowledge, attitude and preventive practice by demographic variables

Characteristics	Mean scores (SD)		
	**Knowledge**	**Attitude**	**Practice**
**Age groups (years)**			
18–30	46.32 (5.92)	34.85 (5.36)	21.89 (2.98)
31–40	44.77 (6.53)	33.62 (5.64)	22.57 (2.78)
41–50	45.37 (6.24)	32.56 (4.75)	22.97 (2.07)
51–90	44.09 (6.47)	31.85 (5.02)	22.96 (2.73)
**Gender**			
Male	45.06 (6.35)	33.72 (5.48)	22.37 (2.74)
Female	46.03 (5.97)	34.06 (5.29)	22.26 (2.92)
**Region of residence**			
**Greater Accra**	46.48 (5.98)	35.28 (5.36)	21.78 (3.29)
Ahafo	45.50 (6.76)	35.25 (7.42)	21.88 (1.25)
Bono East	43.50 (11.22)	36.60 (5.25)	21.70 (2.21)
Bono	44.40 (6.67)	34.34 (6.09)	22.43 (2.36)
Central	45.97 (6.68)	33.80 (6.06)	22.63 (2.79)
Eastern	45.39 (5.18)	34.72 (5.20)	22.43 (3.04)
**Ashanti**	46.31 (5.87)	35.96 (5.89)	21.73 (3.44)
**North East**	46.75 (4.22)	33.92 (3.32)	21.17 (1.27)
**Northern**	47.96 (6.20)	35.74 (5.70)	19.38 (2.81)
Oti region	46.91 (5.52)	33.66 (3.89)	22.58 (2.03)
Savannah	45.58 (6.38)	31.26 (4.93)	22.47 (1.93)
Upper East	46.21 (6.50)	31.68 (6.33)	21.21 (2.51)
Upper West	42.20 (5.53)	31.10 (4.75)	21.80 (2.25)
Volta region	44.29 (6.59)	32.04 (5.07)	23.33 (2.45)
Western north	45.47 (5.88)	32.39 (3.84)	22.19 (1.80)
Western	45.83 (5.98)	33.78 (4.40)	22.59 (2.31)
**Marital Status**			
Never married	46.40 (5.81)	35.10 (5.43)	21.84 (3.01)
Previously marriedǂ	44.24 (7.85)	31.60 (5.71)	22.53 (2.57)
Married/living together	44.79 (6.30)	32.64 (4.91)	22.89 (2.49)
**Work Status**			
Working	45.09 (6.39)	33.41 (5.51)	22.62 (2.68)
Not working	46.20 (5.94)	34.42 (5.21)	21.97 (2.94)
**Employment type**			
Employee	45.39 (6.17)	33.91 (5.37)	22.45 (2.63)
Self-employed	43.82 (7.13)	31.30 (5.61)	23.35 (2.82)
**Type of service**			
Government	45.39 (6.20)	33.73 (5.49)	22.43 (2.51)
Non-government	45.47 (6.08)	34.25 (5.06)	22.57 (2.97)
**Level of education**			
Primary/No formal education	42.69 (8.23)	28.31 (6.49)	23.23 (2.92)
Secondary	45.98 (6.24)	34.33 (5.65)	22.20 (3.12)
Tertiary/Graduate school	45.59 (6.16)	33.89 (5.29)	22.33 (2.77)
**Level of monthly income**			
Less than GHȻ 1000 (< 123USD)	46.06 (5.79)	34.44 (5.30)	21.98 (2.93)
GHȻ 1000 – GHȻ 2,999 (123-371USD)	45.48 (6.45)	33.40 (5.63)	22.55 (2.63)
GHȻ 3,000 – GHȻ 5,999 (372-744USD)	44.69 (6.41)	33.15 (5.43)	22.69 (2.93)
GHȻ 6,000 or more (≥ 745USD)	44.29 (7.22)	33.73 (5.33)	22.90 (2.72)

The mean attitude score towards myopia among the participants was 33.9 ± 5.4 (range, 15 – 48), which declined steadily with an increase in age. Of the 945 participants who were aware of myopia, 424 (45%) had a positive attitude and 520 (55%) had a negative attitude towards myopia. Higher attitude scores were observed among those who were aged 18 to 30 years (34.9 ± 5.4), females (34.1 ± 5.3), and participants who were not married (35.1 ± 5.4). Attitude scores were also higher among participants with secondary level education and lower income earners (< GHȻ 1000 or 123USD), as detailed in Table 2.

The mean score for myopia preventive practices among participants was 22.3 ± 2.8 (range, 12 – 33) but it varied significantly with the demographic variables, as shown in Table 3. Of the 945 participants who were aware of myopia, 57% (*n* = 540) had good myopia preventive practices and the mean scores for protective practices were highest among married people (22.9 ± 2.5) and those who were self-employed (23.4 ± 2.8) but lowest among those living in the Northeast region of Ghana (21.2 ± 1.3).

**Table 3:** One way ANOVA table for myopia knowledge, attitude and preventive practice

Demographic factor	Knowledge		Attitude		Preventive practices	
	F-statistic	*P*-value	F-statistic	*P*-value	F-statistic	*P*-value
Participant age	*F*_(3,941)_ = 5.860	0.001*	*F*_(3,940)_ = 14.986	< 0.001*	*F*_(3,941)_ = 9.419	< 0.001*
Region of residence	*F*_(15,929)_ = 1.791	0.031*	*F*_(15,928)_ = 5.354	< 0.001*	*F*_(15,929)_ = 5.141	< 0.001*
Gender of participant	*F*_(1,943)_ = 8.698	0.003*	*F*_(1,942)_ = 0.890	0.346	*F*_(1,943)_ = 0.382	0.536
Marital status	*F*_(2,942)_ = 8.907	< 0.001*	*F*_(2,941)_ = 29.682	< 0.001*	*F*_(2,942)_ = 15.826	< 0.001*
Work status	*F*_(1,943)_ = 7.493	0.006*	*F*_(1,942)_ = 8.256	0.004*	*F*_(1,943)_ = 12.603	< 0.001*
Employment type	*F*_(1,506)_ = 4.756	0.030*	*F*_(1,506)_ = 18.19	< 0.001*	*F*(_1,506)_ = 9.022	0.003*
Type of service	*F*_(1,414)_ = 0.016	0.900	*F*_(1,414)_ = 0.868	0.352	*F*(_1,414)_ = 0.269	0.604
Education level	*F*_(2,942)_ = 1.688	0.185	*F*_(2,941)_ = 7.513	< 0.001*	*F*(_2,942)_ = 0.812	0.444
Monthly salary level	*F*_(3,941)_ = 2.317	0.074	*F*_(3,940)_ = 3.151	0.024*	*F*_(3,941)_ = 4.293	0.005*

^*^P-value < 0.05 are bolded and indicate statistically significant values

### Differences in mean KAP scores for myopia between demographic variables

Variation in KAP according to respondents’ socio-demographics are presented in Table 3 and the results of the one-way ANOVA for differences in mean KAP scores, showing the F-statistics and their levels of significance. Myopia knowledge varied significantly with most of the demographic variables in this study, including age groups, region of residence, marital status, work status (higher scores for those not working than those who were working) and type of employment (higher for those employed in an institution than for those who were self-employed) (Table 3).

Post hoc analysis revealed statistically significant differences in the mean scores for knowledge among those aged 18–34 years age compared with those aged 31–40 years (mean difference 1.54, 95%CI 0.47–2.62, *P* = 0.005) and people who were aged > 50 years (mean difference 2.23, 95%CI 1.02–3.44, *P* < 0.001). Those who were never married had a significantly higher mean score than those who were either divorced/separated (mean difference 2.16, 95%CI 0.44–3.88; *P* = 0.014) or married (1.61, 0.79, 2.42; *P* < 0.001). The difference in the mean score for knowledge between those who earned less than 1,000 GHC per month and those who earned 3000 – 5,999 GHC (mean difference 1.36, 95%CI 0.01–2.72, *P* = 0.049) was marginally significant. Similarly, participants who lived in the Ashanti region had the highest mean score for knowledge (36.0 ± 5.9), while those who were living in the Upper West region had the lowest mean score (31.1 ± 4.8, Table 2).

The mean attitude scores varied significantly with age groups, marital status, and work status (higher scores for the unemployed), employment type (higher scores for employed vs self-employed), educational level, income level (highest scores among lowest income earners) and place of residence of the participants (*p* < 0.05, for all) but not with gender (*P* = 0.346) or type of service (*P* = 0.868). Post hoc analysis revealed that the younger participants (18-30 years) had a significantly better attitude towards myopia compared with all the other age groups. Those who were never married had higher attitude scores than the previously married (mean difference 3.51, 95%CI 2.04–4.96, *P* < 0.001), and the married participants (mean difference 2.46, 95%CI 1.76–3.15, *P* < 0.001). Participants who had completed secondary education showed a higher mean score than those who had no primary/formal education (mean difference 6.02, 95%CI 2.97–9.07, *P* < 0.001) but had similar scores to those who had completed tertiary education.

For preventive practices, the mean scores varied significantly with the participants’ age, marital status, work status, employment type and region of residence (Table 3). Participants who lived in the Volta region had a significantly higher mean score compared to those in the Greater Accra region (mean difference 1.55, 95%CI 0.99–2.11, *P* < 0.001), Ashanti region (mean difference 1.61, 95%CI 0.97–2.24, *P* < 0.001) and in the Northern region (mean difference 3.96, 95%CI 2.80- 5.11; *P* < 0.001).

### Associations between demographic variables and KAP of myopia in Ghana

Table 4 presents the unstandardized and standardized coefficients for factors associated with the three outcome variables in this study. Region of residence (β = –0.55, 95%CI: -0.87, -0.23), education level (β = 18.35, 95%CI: 14.42, 22.27) and type of service (β = 4.56, 95%CI: 1.22, 7.89) were associated with myopia knowledge in this study. Attitude towards myopia was significantly associated with region of residence in Ghana (β = –0.24, 95%CI: -0.35, -0.14; *p* < 0.001) particularly when comparing between the Greater Accra region and either Volta (mean difference: 3.23, 95%CI: 1.32, 5.15; *p* < 0.001) or Western North regions (mean difference: 2.89, 95% CI: 0.17, 5.61; *p* = 0.022). There were statistically significant associations between preventive practice against myopia and the region of residence (β = 0.07, 95%CI: 0.01, 0.12; *p* = 0.015) and type of employment (β = 2.53, 95%CI: 1.50, 4.91; *p* = 0.037) of the participants in this study. Those who were self-employed demonstrated better practice towards myopia compared to people who were otherwise employed. No other demographic variable was significantly associated with any of the three outcomes in this study (Table 4).

**Table 4:** Standardized coefficients (β) for the demographic factors associated with myopia knowledge, attitude and preventive practices

Variable	**Knowledge**			**Attitude**			**Practice**		
	β	95%CI	*P*-value	β	95%CI	*P*-value	β	95%CI	*P*-value
Participant age	-0.81	-2.43, 1.08	0.449	-0.35	-0.93, 0.24	0.241	-0.01	-0.31, 0.29	0.948
Region of residence	-0.55	-0.87, -0.23	< 0.001*	-0.24	-0.35, -0.14	< 0.001*	0.07	0.01, 0.12	0.015*
Gender of participant	0.02	-3.22, 3.26	0.991	-0.25	-1.28, 0.78	0.634	0.10	-0.43, 0.63	0.715
Marital status	-0.62	-2.64, 1.40	0.545	-0.54	-1.21, 0.13	0.114	0.25	-0.10, 0.59	0.156
Employment type	1.34	-4.11, 6.78	0.839	-1.95	-6.58, 2.67	0.407	2.53	0.15, 4.91	0.037*
Type of service	4.56	1.22, 7.89	0.007*	0.01	-1.09, 1.11	0.983	-0.22	-0.79, 0.34	0.441
Education level	18.35	14.42, 22.27	< 0.001*	-0.31	-2.33, 1.71	0.763	-0.66	-1.70, 0.39	0.216
Monthly salary level	-1.78	-3.95, 0.41	0.112	-0.14	-0.82, 0.55	0.698	-0.16	-0.51, 0.20	0.379

^*^P-value < 0.05 are bolded and indicate statistically significant values

## Discussion

Myopia has long been a public health issue globally and understanding the knowledge, attitudes, and preventive practices (KAPs) related to myopia are important for promoting good vision and the general health of the population. In this study, participant demographics were different since the study was conducted across different regions such that different sub-populations may be exposed to possible varied sources of myopia information. Our results showed significant variations in the myopia KAP scores with most of the demographic variables including age, gender, marital status, work status, employment type, level of education, monthly salary level, and regions of residence. This finding implies that demographic factors are key indicators to be considered when developing any myopia awareness tool. Similar results have been reported in other studies [20], with some also showing contrasting results where no association was found between participants’ demographics and their decision to seek ortho-k or daily-wear soft contact lenses [14].

Myopia has been known over the years to be the most common type of refractive error as well as the leading cause of visual impairment and preventable blindness across the globe [21]. In assessing the awareness and knowledge of myopia among the study population, it was found that less than half of the participants had previously heard about myopia. In the segregated data, the major sources of awareness were visits to hospitals (41.9%) and schools (32.2%). The awareness level was low, given that similar studies in Singapore [22] and Saudi Arabia [23] found about 80.0% and 82.0% of participants were aware of myopia. However, these studies measured awareness in persons with refractive error. Similar studies in Singapore [24], India [25], Pakistan [26] and Kenya [20] have shown that awareness does not necessarily translate to knowledge about myopia or its complications, as demonstrated in a study by Almujalli et al. [23]. In this study, over a quarter of the participants who were aware of myopia still showed low knowledge of myopia.

Myopia progresses steadily with age [27], so one would expect that awareness and knowledge would also increase with age. However, this was not the case in the present study because of the decline in myopia knowledge observed with a decrease in the participants’ age (β = –0.138, 95%CI –1.501, –0.123; *p* = 0.021). Considering the ease with which young people can access various information sources including social media and other internet-based platforms, it is not surprising that young people demonstrated better knowledge of myopia compared with the older people in this study. Similar to our findings, Muma and Oboyo [20] reported that younger age was significantly associated with knowledge of myopia risk factors and corrective measures [20]. Another study in Kenya found similar results indicating that myopia knowledge scores decreased with an increase in the age of the participants [20]. The current findings are in contrast with a KAP study from Ethiopia [28] which reported that awareness and knowledge of refractive errors increase with age. The relatively low awareness of myopia in this study population overall, may be attributed to the poor eye care seeking behaviour and utilisation of eye care services, with women known to utilise health care services more than men [29, 30].

There were regional differences in the myopia knowledge scores among participants, which can be explained by the greater availability and access to health care services in the Northern regions of Ghana. Thus, the Northern region participants have good knowledge, possibly due to the presence of NGOs offering refractive error (RE) services. The knowledge of those participants is comparable to the knowledge of those in urban areas where RE services are readily available.

The somewhat average attitude scores among the participants in this study reflected the knowledge level of myopia in this study population, which may underpin wrong beliefs and attitudes towards myopia and spectacle wear. This is similar to a previous study that showed only 46% of their study participants considered myopia to be a health risk, while another 46% considered it to be an optical inconvenience [12]. Almujalli et al. also reported that 45% of students had a negative attitude towards myopic correction, 35% had a positive attitude and 20% were indifferent [23]. Population-based studies have shown that there is a high prevalence of false beliefs and attitudes toward refractive errors in general and spectacle use, were mostly informed by the sociocultural context. In rural areas, there are beliefs that poor vision is part of the ageing process and that wearing spectacles can damage the eyes, lead to dependence, or worsen eyesight. There is also a high prevalence of stigmatization, fear of being labelled as disabled or handicapped, and cost issues [31, 32]. In more affluent societies, myopia may be regarded as a sign of intelligence, but myopic correction is considered an inconvenience and a discomfort [12].

**Fig. 2:**
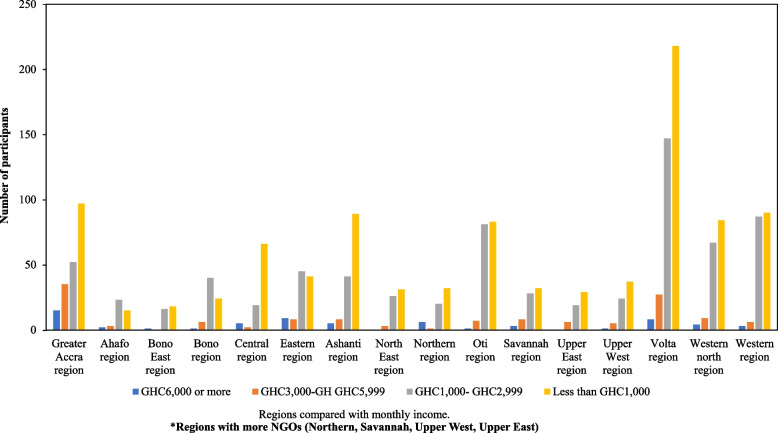
Regions compared with monthly income. *Regions with more NGOs (Northern, Savannah, Upper West, Upper East)

In the current study, attitude scores were higher among participants with secondary and tertiary level education when compared with those with lower levels of education. Educational level is an established social determinant of health and has been shown to potentially affect health through knowledge, health behaviours and socioeconomic status, amongst others [33, 34]. In a study in Saudi Arabia, a large proportion of school children aged 7–14 were aware of myopia but showed negative attitudes towards eyeglass users, and consequently, this led to many of the children with myopia not wearing their glasses [23]. Similarly, significant differences in altitude were also observed between regions in this study, such that participants who lived in the more urbanized regions or regions with a significant presence of NGOs providing health care, recorded higher mean scores compared to less developed regions. Similarly, those in the low-income bracket had higher knowledge and attitude scores compared to people in the high-income bracket which can be attributed to the fact that the low-earning people in our further stratification (Fig. 2) happen to live in the regions with NGOs providing refraction services. Being poor and knowing they might never get a better opportunity; they avail themselves of those services and get the management they need. Higher earning people in the same neighbourhoods would refuse to avail themselves of the services of the NGOs because of the social stigma associated with that [35] and therefore, they remain ignorant. It is also possible that with the advent of COVID-19 and its associated working-from-home practice, many employees were involved in near and screen-based tasks and accompanying increased access to information whereas employers probably played more supervisory roles possibly requiring intermediate to distant visual demands. Other studies identified history of spectacle use, history of eye examination, health education or training on eye health and older age as positively associated with a good attitude [28]. Again, the socio-demographic differences in attitude are consistent with the findings on knowledge, demonstrating the impact of education on behaviour and attitude to health care [28].

In contrast with earlier findings on knowledge and attitude, we found that those that had good knowledge of myopia and a positive attitude, were also less likely to show good preventive practice. These findings indicate that persons who are aware and knowledgeable about myopia, perhaps due to inaccessibility to refractive services or may be less likely to engage in preventive practices. These results underline the importance of public education to correct common public misperceptions and misconceptions, especially regarding health conditions. A study among Saudi women university students also found that participants had general knowledge about refractive error correction methods but more than half of them had never gone for an eye examination or only been examined once or twice in their lifetime [36]. Again, the desire for new information and experience is found to be keen among younger persons [37] and the majority of young people in Gondar city, Northwest Ethiopia agreed that visual impairment from refractive error can be prevented [28]. Although Hafeez et al. (2019) found a significant relationship between preventive practice and educational status, 46.6% of their study population did nothing to prevent myopia [26]. It may also imply that having myopia knowledge may not necessarily translate into myopia preventive practices. Therefore, it may be necessary to provide detailed information regarding the adoption of myopia preventive practices while educating people on myopia.

In preventing and slowing the progression of myopia, lifestyle changes rather than medical treatments have been reported in population studies to show positive outcomes. In our study, married individuals (who were also likely to be parents) were more positive about myopia prevention than younger and unmarried persons. Studies have reported that parents generally regard myopia as a health risk and are more concerned about myopic progression in their children, and thus, engaged in preventive practices which included limiting screen time, restricting studying/reading and encouraging the performance of outdoor tasks, and orthokeratology [12–14]. Other less conventional preventative methods reported were ocular exercises and Acupuncture [13, 38, 39]. In another study among school children, it was indicated that, myopia could be treated by wearing of eye-glasses (30%), avoiding excess use of electronic devices (46%), good nutrition (4%) and surgery (1.0%), while 19.0% had no idea how myopia was treated [23].

In the current study, the mean preventive scores also varied significantly with the region of residence of the participants, such that those who lived in the Volta region reported higher mean scores and lowest scores for those who lived in the Northern region. In a study by McCrann et al. (2018), younger children from rural backgrounds tended to have less screen time than their older and urban-living counterparts [12]. It was not particularly clear if the less screen time for rural-dwelling children found in the study was deliberate as a preventive measure for myopia or if other factors such as availability and ownership of screened devices played a role. Preventive practices for myopia, especially in children, are crucial in slowing its progression [40] and research has shown that early onset myopia in children, if left unchecked, is more likely (than late-onset myopia) to progress to high myopia in adulthood [41, 42].

In our study, employees scored higher for myopia knowledge and attitudes whereas the self-employed only scored higher for preventive practices. The majority of those who are self-employed in Ghana stay outdoors for long periods either because of them being more mobile like going to visit other businesses or securing goods. Increasing outdoor exposure, even by just two hours a day, has a profound effect on the onset of myopia and may also reduce the progression of myopia [43].

### Limitations and strengths

First, since the main tool for this study was a questionnaire, there were chances for recall and information bias from the respondents. Second, due to the cross-sectional nature of the study, the findings reported in this study does not represent a causal relationship, rather only inferences as to the best explanation could be made, despite strong associations. Third, the questionnaire was self-administered, and therefore dependent on self-reported data, which may be unreliable. Fourth, as the survey was an online-based survey available only in English, it may not have captured the responses from regions with restricted access to social media and the internet as well as those with less educated background who are less likely to be proficient enough in the use of English to have taken part in our study. All these limitations could easily have introduced some bias in the selection, demography, sampling, and coverage of respondents in the study. Overall, this is the first study to provide evidence on such an important public health issue which can be used for designing targeted messages directed at the sub-populations identified. Robust statistical analysis was also employed to establish relationship between variables.

## Conclusions

Ghanaian participants had adequate knowledge of myopia which varied significantly between regions and was modified by socio-demographic factors. They also demonstrated good myopia preventive practices although they largely had a negative attitude towards myopia. While myopia knowledge was predicted by participants’ age, myopia preventive practices were predicted by the type of employment. Participants’ region of residence, however, could predict both myopia attitude and preventive practices. The fact that these participants had higher myopia knowledge scores, but low preventive practice scores suggest that while they were aware of myopia there was a disconnect between that awareness and acting on it to engage in preventative practices against myopia. There are a few possible reasons for this disconnect. The factors identified in this study should be considered when planning myopia health education tools for public enlightenment campaigns.

### Supplementary Information

**Additional file 1.**

## Data Availability

The dataset supporting the conclusions of this article is included within the article (and its additional files). Data is also available on request from the corresponding author OUL.

## Abbreviations

KAP: Knowledge, Attitude and Preventive practices
SSA: Sub–Saharan Africa
ANOVA: Analysis of variance
NEHP: National Eye Health Programme
CI: Confidence interval
