# Supplementary material for: Demographic factors associated with myopia knowledge, attitude and preventive practices among adults in Ghana: a population-based cross-sectional survey
Source: BMC Public Health. 2023 Sep 4;23:1712. doi: 10.1186/s12889-023-16587-7 (PMC10476336; doi:10.1186/s12889-023-16587-7)
Supplement: Supplementary file 1 — Additional file 1. [file 12889_2023_16587_MOESM1_ESM.docx]

**Supplementary Material**

**Table-S1**: Sample of the questionnaire

**CONSENT OF WILLINGNESS**

By ticking the agree option, you acknowledge that your participation in the study is voluntary, you are 18 years of age or above, and that you are aware that you may choose to discontinue your participation in the study at any time and for any reason.

□Agree □Disagree

**SECTION A: Socio-Demographic Data**

Instruction: Please answer the questions below or tick (√ ) in the appropriate box.

1. What is your age? ______________________
2. Region of residence?

□Ahafo Region □Ashanti Region □Bono Region □Bono East Region

□Central Region □Eastern Region □Greater Accra Region □North East Region

□Northern Region □Oti Region □Savannah Region □Upper East Region

□Upper West Region □Volta Region □Western Region □Western North Region

1. What is your sex? □Male  □Female

□Other (please specify)

1. What is your marital status?  □ Single □Married □Divorced

□Widow/widower □Separated □ Living together

1. Work status □Retired □Employed □Unemployed
   1. If employed, are you □ Self-employed □Employee
   2. If an employee, are you working for the government? □Yes □No
2. What is your occupation? ___________________________________________________________
3. Highest level of education completed:
   1. □ No Formal education
   2. □ Primary
   3. □Secondary
   4. □Tertiary
   5. □Graduate School
4. What is your monthly income level?

□ Below GHȻ200

□ GHȻ 200 – GHȻ 499

□ GHȻ 500 – GHȻ 999

□ GHȻ 1000- GHȻ2,999

□ GHȻ3,000 – GHȻ5,999

□ GHȻ6,000 – GHȻ 10,000

□Above GHȻ 10,000

1. Please choose from options below, how many hours (0-0hr, 1-1hr, 2-2hrs, 3-3hrs, 4-4hrs, 5-5hrs, 6-6hrs, 7-7hrs, 8-8hrs, 9->8hrs) you spend on the following activities each day. NB: There are Ten (10) options for each activity.

| s/n |  | 0hr | 1 hr | 2 hr | 3 hr | 4 hr | 5 hr | 6 hr | 7 hr | 8 hr | >8 hr |
| --- | --- | --- | --- | --- | --- | --- | --- | --- | --- | --- | --- |
| 1 | Reading books, journals, etc. | □ | □ | □ | □ | □ | □ | □ | □ | □ | □ |
| 2 | Reading and typing on a computer, mobile phone, ipads or any other electronic device | □ | □ | □ | □ | □ | □ | □ | □ | □ | □ |
| 3 | Outdoor mechanical equipment | □ | □ | □ | □ | □ | □ | □ | □ | □ | □ |
| 4 | Cooking and cleaning | □ | □ | □ | □ | □ | □ | □ | □ | □ | □ |
| 5 | Walking and/or running outdoors including other sporting activities | □ | □ | □ | □ | □ | □ | □ | □ | □ | □ |
| 6 | Farm work (clearing and weeding grasses) | □ | □ | □ | □ | □ | □ | □ | □ | □ | □ |
| 7 | Farm work (tending to animals) | □ | □ | □ | □ | □ | □ | □ | □ | □ | □ |

**SECTION B: Awareness of myopia**

1. Have you ever heard of myopia (short sightedness)?  If □Yes, Proceed to Q12
2. If ‘Yes’, where did you hear it from?

□School

□Hospital/health facility

□Television □Radio

□Newspaper

□Internet

□Bill Board

□Other (Please specify)

**SECTION C: Knowledge about myopia**

1. What is myopia? □ Long-sightedness □Blindness □Blurry vision □ Short-sightedness

□Other (Please specify)

1. Which of the below listed conditions/activities can increase your chances (risk factors) of having myopia? (Please check all that apply)

Reading a lot □Yes □No □Not sure

Playing outside with friends □Yes □No □Not sure

High blood pressure □Yes □No □Not sure

Using the computer always □Yes □No □Not sure

My parent(s) have it □Yes □No □Not sure

Playing video games, a lot □Yes □No □Not sure

Diabetes □Yes □No □Not sure

Other(s) (Please specify)

1. What are the signs and symptoms of myopia? (Please check all that apply)

Watery eyes □Yes □No □Not sure

Excessive blinking □Yes □No □Not sure

Headaches □Yes □No □Not sure

Squinting □Yes □No □Not sure

Yellowish eyes □Yes □No □Not sure

Itchy eyes □Yes □No □Not sure

Tired eyes □Yes □No □Not sure

Others (Please specify)

1. What is(are) the treatment for myopia? (Please check all that apply)

Wearing spectacles □Yes □No □Not sure

Prescription medicine including eye drops □Yes □No □Not sure

Increase time used in playing on phones □Yes □No □Not sure

Eating lots of carrots □Yes □No □Not sure

Preparing food with plenty palm oil □Yes □No □Not sure

Sitting far away from the television □Yes □No □Not sure

Wearing special contact lenses □Yes □No □Not sure

Others (Please specify)

1. How do you prevent myopia from occurring (especially for children)? (Please check all that apply)

Taking Vitamin A tablets □Yes □No □Not sure

Early diagnoses □Yes □No □Not sure

Playing outdoor/exercise □Yes □No □Not sure

Exposure to sunlight □Yes □No □Not sure

Reduce screen time □Yes □No □Not sure

Other(s)(please specify)

1. For someone already diagnosed with myopia, how do you prevent it from getting worse/progressing? (Please check all that apply)

Increase reading time □Yes □No □Not sure

Use spectacles □Yes □No □Not sure

Reduce screen time □Yes □No □Not sure

Medication □Yes □No □Not sure

Vitamin A □Yes □No □Not sure

Use special contact lenses □Yes □No □Not sure

Other(s) (Please specify)

1. **Please tick in the box your level of agreement (1-Strongly disagree, 2-Disagree, 3-Somewhat disagree, 4-Somewhat agree, 5-Agree, 6-Strongly agree) with the following statements about myopia. NB: There are Six (6) options for each statement.**

|  | **1** | **2** | **3** | **4** | **5** | **6** |
| --- | --- | --- | --- | --- | --- | --- |
| Children cannot have myopia | □ | □ | □ | □ | □ | □ |
| Myopia only affects albinos | □ | □ | □ | □ | □ | □ |
| Anyone can have myopia | □ | □ | □ | □ | □ | □ |
| Myopia is a genetic disorder | □ | □ | □ | □ | □ | □ |
| Diabetes is a major risk factor for myopia | □ | □ | □ | □ | □ | □ |
| Myopia only occurs in old people | □ | □ | □ | □ | □ | □ |
| There is no cure for myopia | □ | □ | □ | □ | □ | □ |
| Spectacles can prevent myopia | □ | □ | □ | □ | □ | □ |
| Wearing spectacles make your eye weaker and condition gets worse | □ | □ | □ | □ | □ | □ |
| Myopia only affects those with high blood pressure | □ | □ | □ | □ | □ | □ |
| Myopia can be prevented | □ | □ | □ | □ | □ | □ |

**SECTION D: ATTITUDES ABOUT MYOPIA**

1. Please indicate how much (1-Strongly disagree, 2-Disagree, 3-Somewhat disagree, 4-Somewhat agree, 5-Agree, 6-Strongly agree) you agree or disagree with the following statements. NB: There are Six (6) options for each statement.

| **Statement** | **Strongly agree** | **Agree** | **Somewhat agree** | **Somewhat disagree** | **Disagree** | **Strongly disagree** |
| --- | --- | --- | --- | --- | --- | --- |
| Eye health should be taken seriously | □ | □ | □ | □ | □ | □ |
| Myopia is not too common around here | □ | □ | □ | □ | □ | □ |
| I don’t need spectacles for blurry vision | □ | □ | □ | □ | □ | □ |
| I expect to have good sight all my life | □ | □ | □ | □ | □ | □ |
| Wearing sunshades protects the eyes | □ | □ | □ | □ | □ | □ |
| People diagnosed with myopia recover fully with time | □ | □ | □ | □ | □ | □ |
| People who wear spectacles do so for fashion not health | □ | □ | □ | □ | □ | □ |
| People diagnosed with myopia have weak genes | □ | □ | □ | □ | □ | □ |

1. Has a doctor or health professional ever told you that you have the following conditions and/or diseases?

Myopia  □ Yes □ No Astigmatism □ Yes □ No

High blood pressure □ Yes □ No Diabetes □ Yes □ No

Cataracts □ Yes □ No

Other eye health issue (Please specify)

**SECTION E: PREVENTIVE PRACTICES**

1. **Please indicate how much (1-Never, 2-Sometimes, 3-Often, 4-Always) you agree with the following statements. NB: There are Four (4) options for each statement.**

| **Statement** | **Always** | **Often** | **Sometimes** | **Never** |
| --- | --- | --- | --- | --- |
| I read for long hours at a time | □ | □ | □ | □ |
| I get sufficient sleep (6-8 hours) every night | □ | □ | □ | □ |
| I use my phone or tablet for long periods of time | □ | □ | □ | □ |
| I make sure to get some outdoor activities (walking, gardening, spending time outside) done on a daily basis | □ | □ | □ | □ |
| I use the ‘dim’ function on my electronic device(s) | □ | □ | □ | □ |
| I eat foods rich in vitamin A (Green leafy vegetables, yellow sweet potatoes, Carrots, palm oil, etc) | □ | □ | □ | □ |
| I use sun shades when I am outdoors during the day | □ | □ | □ | □ |
| I use electronic devices even in dim light | □ | □ | □ | □ |
| I take intermittent breaks from my computer or phones | □ | □ | □ | □ |

Supplementary material (S2)

**Full Consortium member list**: Centre for Eyecare & Public Health Intervention Initiative (CEPHII)

Uchechukwu L. Osuagwu

^1^Bathurst Rural Clinical School (BRCS), School of Medicine, Western Sydney University, Bathurst, NSW 2795, Australia. African Vision Research Institute, Discipline of Optometry, University of KwaZulu-Natal, Westville Campus, Durban, 3629, South Africa

Kingsley E. Agho

^7^School of Science and Health, Western Sydney University, Campbelltown, NSW 2560, Australia. African Vision Research Institute, Discipline of Optometry, University of KwaZulu-Natal, Westville Campus, Durban, 3629, South Africa

Kelechi C. Ogbuehi

^10^Department of Medicine, Dunedin School of Medicine, University of Otago.

Stephen Ocansey

^2^Department of Optometry and Vision Science, School of Allied Health Sciences, College of Health and Allied Sciences, University of Cape Coast, Ghana.

Sylvester Kyeremeh

^4^Department of Optometry and Visual Science, College of Science, KNUST

Nana Darkoah Nkansah

^12^ Koforidua Regional Hospital, Koforidua, Eastern Region, Ghana.

Godwin Ovenseri-Ogbomo

^5^Department of Optometry, Centre for Health Sciences, University of the Highlands and Islands, Inverness, IV2 3JH, UK

Antor O. Ndep

^3^Health Education & Health Promotion Unit, Department of Public Health, Faculty of Allied Medical Sciences, College of Medical Sciences, University of Calabar, Cross River State, Nigeria.

Bernadine N. Ekpenyong

^6^Epidemiology and Biostatistics Unit, Department of Public Health, University of Calabar, Calabar, Nigeria.

Edgar Ekure

^8^Salus University Pennsylvania, USA.

Khathutshelo Percy Mashige

^9^African Vision Research Institute, Discipline of Optometry, University of KwaZulu-Natal, Westville Campus, Durban, 3629, South Africa

Kovin Shunmugan Naidoo

^13^School of Optometry and Vision Science, University of New South Wales, Sydney, New South Wales, Australia.

Jyoti Naidoo

^14^School of Optometry and Vision Science, University of New South Wales, Sydney, New South Wales, Australia

Tuwani Rasengane

^11^Department of Optometry, University of the Free State and Universitas Hospital, Bloemfontein, South Africa.
